# Low-income parents’ perceptions of the importance of a musical training programme for their children: a qualitative study

**DOI:** 10.1186/s12889-020-09568-7

**Published:** 2020-09-25

**Authors:** Laurie Long Kwan Ho, William Ho Cheung Li, Ankie Tan Cheung, Wei Xia, Ka Yan Ho, Joyce Oi Kwan Chung

**Affiliations:** 1 School of Nursing, University of Hong Kong, 21 Sassoon Road, Pokfulam, Hong Kong SAR; 2 School of Nursing, the Hong Kong Polytechnic University, Hung Hom, Hong Kong SAR

**Keywords:** Parents’ perceptions, Musical training, Preschool children, Poverty, Psychological health, Parent-child relationship, Low-income families

## Abstract

**Background:**

Despite clear evidence for the effectiveness of musical training in promoting psychological well-being among underprivileged children, parents’ perceptions of the importance of such training for their children remains unknown.

**Methods:**

Of the parents of 171 underprivileged preschool children in Hong Kong who had participated in a free musical training programme, 25 were randomly selected and invited to participate in individual semi-structured interviews. Colaizzi’s descriptive phenomenological data analysis strategy was followed for analysing the data.

**Results:**

The results showed that parents identified numerous benefits of the programme for their child, including increased happiness, improved confidence, positive behavioural changes, and enhanced parent-child relationships. At the beginning of the programme, parents tended to disregard the usefulness of musical training but gradually came to recognise its importance for their children’s psychological and social well-being. However, children were limited by their parents’ financial constraints from participating in musical training after the free programme ended.

**Conclusions:**

These findings imply that existing policy may overlook the psychosocial needs of underprivileged children and suggest that more resources should be allocated to facilitate the continuity and sustainability of such a free programme for this vulnerable population.

**Trial registration:**

ClinicalTrials.gov NCT02762786, registered on May 5, 2016.

## Background

Poverty can have detrimental effects on children’s psychological and social well-being as a result of the long-term adversity it imposes throughout childhood [1–3]. Evidence suggests that underprivileged preschool children are more prone to psychological and social problems than their counterparts from high-income families. For example, several studies have shown that approximately 25% of underprivileged preschool children suffer from varying degrees of issues such as low self-esteem, depressive symptoms, and poor social skills [4–7]. Additionally, a systematic review showed that underprivileged preschool children were five times more likely to have behavioural problems than those in other age groups [7]. They also exhibited weaker adaptive abilities later in life, making them more vulnerable to serious emotional and behavioural problems during adolescence and adulthood [7, 8].

As early interventions for underprivileged preschool children have been found to promote psychological and social well-being, as well as to prevent behavioural problems in later life [4, 9], it is of paramount importance that healthcare professionals develop and implement appropriate interventions targeting this population. There has been an increased use of musical training to promote mental health of children and adolescents [10], to enhance quality of life and reduce depressive symptoms in paediatric brain tumour survivors [11] and to improve social skills of children with autism [12]. Previous studies have found that active engagement in musical activities has beneficial effects on quality of life and psychosocial well-being by provoking positive emotions and cognitive and social development [13–16]. Growing evidence indicates that music education programmes promoted psychological well-being, social inclusion and cognitive development in infants, school-aged children and youth [17–21]. However, there is a lack of empirical evidence on the effectiveness of musical training for preschool children. One of the few studies to examine the effectiveness of a musical training programme for underprivileged preschool children, was a quasi-experimental study our team conducted in 2018 [22]. The study showed that such training could increase participants’ happiness and enhance their quality of life [22]. Yet, this study did not elicit parents’ perceptions of their children’s musical training.

A few studies conducted in Hong Kong revealed that underprivileged families were unable to afford extra-curricular activities for children aged 10–13, and thus some parents regarded musical training as a luxury and an optional leisure activity without any therapeutic benefits [5, 6]. Understanding parents’ perceptions is a crucial step in facilitating the continuity and sustainability of an effective musical training programme that can promote the psychological and social well-being of their children. This study aimed to collect in-depth qualitative data about parents’ perspectives on a musical training programme and its influence in the family context.

## Methods

### Study design and sample

A qualitative study using a phenomenological approach was conducted from August to October 2018. Parents (*n* = 100) whose children attended the previously mentioned quasi-experimental study were eligible for participation. The study examined the effectiveness of a 12-week free musical training programme among underprivileged Chinese preschool children in Hong Kong [22]. Detailed study methods are described elsewhere [22]. Briefly, a weekly 1-h musical training programme was implemented in small groups with approximately 10 children per group without parents’ participation. The programme was delivered by professionally qualified musicians who had extensive experience in early childhood music education. The musical training intervention was designed to stimulate children’s awareness of music and allow them to experience music-making by singing, dancing, body percussion and exploration of musical instruments. As mentioned previously, the programme found improved happiness and quality of life defined as measures of subjective well-being, including assessment of life and its components such as physical ability, psychological state, social relationships and school functioning [23, 24]. Participants were randomly selected by a computer program upon the completion of the musical training programme. Final sample size of this study was determined by data saturation, which was achieved after interviewing 25 parents with 100% response rate.

### Data collection

Ethical approval was obtained from the Institutional Review Board of the University of Hong Kong/ Hospital Authority Hong Kong West Cluster. Written consent was obtained from parents after they were told the purposes of the study and had agreed to participate. The participants were informed that the study was completely voluntary and were assured of data confidentiality. They were also informed that they could withdraw from the study at any time with no prejudice.

Participants were invited to attend an individual semi-structured interview in a private meeting room. The interviews were audio-taped and lasted from 20 to 30 min. Field notes were taken throughout the interviews. All of the interviews were conducted by two qualified nurses who had extensive experience in conducting qualitative interviews and received training from an associate professor with expertise in paediatrics. One nurse played the role of an interviewer and encouraged the participants to express their feelings and thoughts freely and honestly. The other nurse worked as an observer and sought to detect participants’ nonverbal cues such as facial expressions and body gestures.

A semi-structured interview guide was developed by a group of qualitative and paediatric research experts, including an associate professor, an assistant professor, and a postdoctoral fellow. Expert advice was also sought from a professional musician who has acquired over 20 years of experience in music education for children. The interview guide was designed to cover two major areas: 1) perceptions of change in one’s child or family after that child’s participation in the musical training programme, and 2) perceptions of the importance of the musical training.

The interviews began with a broad and open question, for instance, “Can you share something about your family background such as the monthly income or the living environment?” This was followed by nondirective questions related to music (eg, “What are your perceptions of music?”) and related to musical training programmes (eg, “What do you think about the musical training programme?” or “How do you evaluate the usefulness of the musical training?”). Different probing techniques (eg, “Can you give me some examples?”) were applied throughout the interviews to elicit more detailed and comprehensive information. An additional file shows this in more detail (see Additional file 1).

### Data analysis

Colaizzi’s descriptive phenomenological data analysis strategy was followed for analysing the data [25]. To accurately capture the content of the dialogue and physical expressions that took place during the interview, all recordings were transcribed verbatim into Cantonese immediately after the interviews. Important quotes relevant to the emerging themes were identified and translated into English for the purpose of reporting. Two researchers who were experienced in qualitative and paediatric research for about 5 years were responsible for coding. They analysed the data independently and then compared their results, a process that helps ensure stability and consistency of the findings. To improve objectivity and reduce personal bias, the two researchers recorded their data analysis procedures and documented their reflections on it from time to time. All of the transcripts were reviewed intensively to identify statements that were relevant to the phenomenon under investigation (i.e. parents’ perspectives on a musical training programme and its influence) and labelled as significant statements. The identified meanings that were common across the transcripts were then grouped into categories and themes by examining their similarities. By organizing all the themes, a full and inclusive description of the phenomenon was emerged. The nonverbal behaviours and interactions from field notes provided additional details about participants’ feelings which aided the data analysis.

Quality and rigour—in terms of credibility, transferability, dependability, and confirmability—was achieved through a number of strategies. For example, triangulation strategies were adopted to enhance credibility; these included taking field notes throughout the interviews to capture supplementary, nonverbal cues, and having two researchers involved in data analysis [26]. These methods helped ensure consistency of the data through multi-method data collection, and achieve more comprehensive data analysis. Credibility was further enhanced with member checking to validate results with all participants themselves [26]. All participants in our study showed agreements with our results. Participants were also encouraged to express their feeling and ideas honestly throughout this stage. Confidentiality was assured, and privacy was provided throughout the interviews by offering a safe and secure environment for participants. Transferability was enhanced by using direct quotes and explicit descriptions of participants’ experiences, and is illustrated in the similarities drawn between these and other research findings. The technique of stepwise replication was adopted to achieve dependability [27]. Moreover, to maintain further consistency, all the interviews were carried out by the same researchers. Finally, extended reflection on the data analysis process was implemented by reviewing the documented procedures and reflections at regular interval to improve confirmability of the results [27]. Research team meetings were held at regular intervals to monitor the data analysis process and manage any divergence of views by aggregating expert opinions to ensure data stability and consistency.

## Results

### Participant characteristics

Participant demographics are shown in Table 1. Most (84%) of the participants were mothers (Mean age = 35.8 years). More than 50% of the participants were living in subdivided urban flats. Children of all the participants (Mean age = 4.7 years) finished the entire course of the musical training programme between March and August 2018.

**Table 1:** Participant demographics in the semi-structured interviews (*N* = 25)

	Frequency (%)
**Sex**	
Male	4 (16)
Female	21 (84)
**Marital status**	
Married	19 (76)
Divorced/ separated	6 (24)
**Educational level**	
Primary	1 (4)
Lower secondary	10 (40)
Higher secondary	12 (48)
Tertiary	2 (8)
**Employment status**	
Employed	8 (32)
Unemployed	17 (68)
**Types of housing**	
Subdivided flats	13 (52)
Public rental housing	11 (44)
Private rental housing	0 (0)
Private housing (owner)	1 (4)
**Previous musical experiences**	
Yes	2 (8)
No	23 (92)
**Mean (SD)**	
**Age (parents), yr**	35.8 (5.9)
**Age (children), yr**	4.7 (1)

We identified three main themes: (1) perceptions of music and musical training; (2) perceived beneficial effects of musical training programme; and (3) the difficulty of sustaining musical training for one’s child. Several subthemes were further identified under each main theme.

#### Perceptions of music and musical training

The perceptions held by the participants seemed to affect their reactions towards music and musical training. Three subthemes were identified under this theme.

##### Music is important but not a necessity

In general, the majority of participants agreed that music might have beneficial effects for the psychological well-being and the development of their child. However, they also shared the similar view that music is not a necessity.

*Music is somehow important. I believe listening to music can soothe our souls and make us feel good. However, I do not have time to sit down and enjoy the music. I need to work very hard to support the family. Listening to music cannot relieve my workload. (Mrs. Y)I think music is somehow good for my son’s growth and development. However, I believe that food is more important than music for the growth and health of my son. (Miss. C).*

##### Changing perceptions of musical training

Most of the participants admitted that when they first signed their child up for the musical training programme, they disregarded its potential usefulness. Not until their child had participated in the programme and exhibited positive changes did the parents’ perceptions of the programme undergo a significant alteration.

*I realized that the musical training programme changed something about my son; he looked happier than before, which is something I could never have imagined. (Mr. W).*

*In the beginning, I just thought it was good to have something free for my daughter’s entertainment. I did not expect that the programme would be good for her psychological well-being. (Mrs. Q).*

##### The free musical training is a valuable opportunity

All of the participants mentioned their amazement at having a free musical training programme for their child, and stated that they did not hesitate to make use of the opportunity to let them join.

*The musical training programme provides a valuable opportunity for my son to learn music. (Miss. C).*

#### Perceived beneficial effects of the musical training programme

##### Increased happiness

Many of the participants observed positive mood changes in their children after they participated in the musical training programme. Since their children had started joining the programme, some found that their children were less likely to lose their temper and cry, and were happier when they were at home.

*My son cries about many things, such as asking me to take him outdoors to play. He is an active boy and he often complains that it is too boring to stay at home. But, you know, I am a single mother and need to take care of him by myself. And I really do not have extra money and time to buy him toys or take him out to play. However, after joining the musical training programme, he knows that he will have a lesson once per week so he stops whining to go out. (Mrs. B).*

*My daughter loves attending the musical training programme. She has changed a lot since taking part in the program and is happier than before. (Mrs. Q).*

##### Improved confidence

A number of participants mentioned that their child was too shy to dance and sing at first. However, they were surprised that their child was able to perform singing and dancing on the stage by the end of the musical training programme.

*My son is introverted. I know he is affected by the fact that he comes from a single-parent family. I know he cares much about the fact that he doesn’t have a father … I was so amazed and impressed to see my son confidently performing what he had learnt from the musical training programme on the stage. (Miss. E).*

##### Improved concentration

The majority of participants noted an improvement in their child’s concentration after joining the musical training programme. Most of the families lived in subdivided flats, and mentioned that their living environments were very crowded and noisy while also describing their child was easily distracted by the surroundings. Parents observed that, since their child had cultivated the habit of listening music after joining the programme, their child had become more focused and was capable of attending to a single task rather than being distracted by their surroundings.

*We live in a subdivided flat that is so small that we can hear all of the sounds from the neighbours. Prior to the programme, my son was often distracted by the environment. Now, he likes to listen music and do homework by himself. I think the musical training programme has cultivated his interest in listening to music and this hobby makes him more able to concentrate. (Mrs. H).*

*It is very difficult to ask my son to concentrate on a single task. For example, he would suddenly go to draw or play puzzles while he was doing homework. I am so impressed that he is now able to sit quietly and enjoy listening to music at home. Sometimes, he practises singing and dancing that he has learned from the musical training programme by himself. (Miss. C).*

##### Promoting independence and social relationships

Many participants mentioned that, to limit their expenses, they seldom took their child out. This made their child very dependent on them and increased the likelihood that their child would exhibit difficulties getting along with other children. The parents considered the free musical training programme an opportunity for their children to learn how to get along with others. Some participants also mentioned that their child learned some social manners, such as how to line up and follow instructions.

*We seldom go out and my daughter spends most of her time with me at home. This may make her dependent on me and fearful of strangers. She cried at first in the musical training programme but she eventually adapted to the separation. (Mrs. I).*

*At the beginning, I was worried about whether my daughter would get along with other children because she is the only child at home. However, I was very surprised when she introduced her best friend to me last week. (Mrs. F).*

##### Enhanced parent-child relationships

The musical training programme appeared to have elicited positive changes in parent-child relationships by increasing parent-child interactions. The participants reported that the programme had provided a new topic to talk about with their child; their children were excited to share the things that happened in their lessons and some even taught their parents how to dance. The participants reported spending more time with their children and fostering a better parent-child relationship after the programme.

*We have become closer than before. My son always tells me about the things that happened during the lesson. He holds my hand and teaches me how to dance. We have a good time with each other. (Miss. J).*

*My daughter spends more time chatting with me. She likes to share things that she has learned from the musical training programme with me. (Mr. V).*

#### Difficulty sustaining musical training for one’s child

Most participants mentioned that they did not have extra money to spend on extra-curricular activities, such as music classes, for their child. Although they understood that musical training could benefit their child, they could not afford to pay for their child’s enrolment after the free training programme ended.

*I know my daughter likes to participate in the musical training programme very much. However, I do not have extra money for such a purpose. (Mr. V).*

*As we cannot afford the musical training fee, I really hope that the non-government organisation can continue offering a free musical training programme for my daughter. (Miss. J).*

*I agree that the musical training programme is good for my son and I know that learning music is a common practice for children in Hong Kong. But how can we afford it? (Miss. N).*

## Discussion

To the best of our knowledge, this is the first study to explore how low-income Hong Kong Chinese parents perceive the importance of musical training for their preschool children. In addition to an increased level of happiness, which the previous experimental study [22] also observed, this study showed that these parents perceived many other benefits of the training programme for their child, including improved confidence, observable positive behavioural changes (i.e. development of independence and social manners), and enhanced parent-child relationships. Our findings are consistent to previous studies that have also examined community-based music programmes targeting children in other age groups [17–19]. Parents in this study perceived more benefits than previous studies which may be due to the combined different components of music in our musical training programme including singing, dancing and exploration of musical instruments [20, 21].

Our findings show that most of the parents overlooked the potential beneficial effects of musical training on the psychological and social well-being of their children before joining the programme. This also explained why we found it difficult to recruit this population for participating in the musical training programme. The findings suggest that when promoting musical training to low-income parents, more and better information should be given to highlight the benefits for their children. For example, information leaflets and videos could be provided. Indeed, although most parents in the present study perceived the benefits of the musical training after joining the programme, they emphasised their inability to pay for extra-curricular activities for their children, especially continuous musical training. More resources should be allocated to facilitate the continuity and sustainability of such free programmes for underprivileged children. Importantly, lack of recreational activities has been strongly associated with poor psychological and social well-being, and a higher risk of suffering from depression in underprivileged children [5, 6, 28]. The free musical training programme, which is a leisure activity that has been found to be effective to promote underprivileged children’s overall well-being and quality of life [22], might be an appropriate and useful strategy to ameliorate such detrimental effects of poverty.

Poverty has been strongly associated with negative parenting practices that consequently hinder parent-child relationship [29–31]. In this study, parents claimed that the musical training programme fostered positive parent-child relationships by promoting interpersonal interactions. The findings show that children shared the things that happened during their music lessons with their parents, and some even taught their parents how to dance. This finding aligns with previous studies in which children aged 5–12 years who joined music groups spent more time talking with their parents [32, 33]. Jacobsen et al. [33] suggests that this can lead to a stronger sense of self-perceived parental competency in communicating with their children, and to a greater number of constructive parent-child conversations. Positive relationship formation has also been found to contribute to some social benefits associated with higher levels of self-esteem in children [32].

The findings of this study suggest that musical training is an important activity for fostering positive behaviour in children. At the same time, it can help prevent the development of behavioural problems, which is consistent with the findings of previous studies [34, 35]. Parents in this study mentioned that their children demonstrated personal development and increased social adjustment after participating in the musical training programme, such as being more independent and more willing to get along with others. It can be argued that these positive developments in children contribute to changes in parents’ perception towards musical training programmes.

### Limitations

This study had several limitations. First, it appears that mothers were more willing to participate than fathers. A gender difference in perceptions about the impact of the programme may affect our results. The involvement of both fathers and mothers is recommended for future studies. Second, we used proxy informants (i.e., parents) in this study because the children involved in the musical training programme were too young to provide reliable information. Third, we used the self-report data collection method that was susceptible to social desirability bias, especially for an underprivileged population [36].

### Implications for practice

This study enhanced our understanding of parents’ perspectives on the importance of a musical training programme for their children. The qualitative approach further explains and evaluates the findings of the previous experimental study in a real-life context [22]. In particular, these parents’ perceptions of the importance of the musical training programme might encourage wider adoption and maintenance of regular musical training in the future.

Given the potential psychological and social benefits to underprivileged children of a musical training programme, healthcare professionals, non-governmental organizations (NGOs) and schools should explore coordinated efforts to promote such programmes at the community level. Our findings also increase awareness about the involvement of music educators in programmes which aim at promoting children’s psychological well-being. Additionally, increased governmental resources, particularly financial support, allocated to underprivileged children, would improve and widen the opportunities for those children to receive musical training as a way to promote their psychological and social well-being. Ultimately, the risk of underprivileged children developing psychiatric illnesses as a result of the adverse effects of poverty may be mitigated by this community programme. Our musical training programme can be a reference for other countries and regions to promote early childhood music education.

## Conclusion

This study addressed a gap in the literature by soliciting low-income Hong Kong Chinese parents’ perspectives on the importance of a musical training programme for their preschool children. The findings revealed parents’ perceptions of musical training changed; in particular, they realized the importance of such training in promoting their children’s psychological and social well-being. We suggest medical professionals and NGOs should explore coordinated efforts to promote such free programmes at the community level especially for this vulnerable population.

## Supplementary information

**Additional file 1.** Interview guide. An interview guide for semi-structured interviews.

## Data Availability

The datasets generated and/or analysed during the current study are not publicly available due to privacy or ethical restrictions but are available from the corresponding author on reasonable request.

## Abbreviation

NGOs: Non-Governmental Organizations
