# Supplementary material for: Low-income parents’ perceptions of the importance of a musical training programme for their children: a qualitative study
Source: BMC Public Health. 2020 Sep 25;20:1454. doi: 10.1186/s12889-020-09568-7 (PMC7519511; doi:10.1186/s12889-020-09568-7)
Supplement: Supplementary file 1 — Additional file 1. Interview guide. An interview guide for semi-structured interviews. [file 12889_2020_9568_MOESM1_ESM.docx]

**Interview guide**

| Introduction | - Greeting - Explain the study purpose - Ensure completely voluntary and data confidentiality - Ensure they have the right to withdraw from the study at any time with no prejudice - Provide privacy |
| --- | --- |
| Beginning | (Open question)   - Can you share something about your family background such as the monthly income or the living environment? - How is your daily life with your children? |
| Major area relevant to the phenomenon under investigation | 1. Perceptions of change in children or family after participating in the musical training programme   - What are your perceptions of music? - For you, what is music? Have you learnt music before? - How important is music in your life/ for your children?   2. Perceptions of the importance of the musical training   - What do you think about the musical training programme? - What are your perceptions of musical training? - How do you evaluate the usefulness of the musical training? - Is there any difference/ change you have noticed after your children joining the musical training programme? If yes, what is it? - How the changes affect your /your children’s daily life? - How the changes affect the relationship between you and your children? - Do you think you will allow your children to join the musical training in the future? |
| Ending | - Thank you for their participation - Ask if they have any questions |
